# Hypoxic Signaling During Tissue Repair and Regenerative Medicine

**DOI:** 10.3390/ijms151119791

**Published:** 2014-10-31

**Authors:** Tessa D. Nauta, Victor W. M. van Hinsbergh, Pieter Koolwijk

**Affiliations:** 1 Department of Physiology, Institute for Cardiovascular Research, VU University Medical Center Amsterdam, Van der Boechorststraat 7, Amsterdam 1081 BT, The Netherlands; E-Mails: t.nauta@vumc.nl (T.D.N.), v.vanhinsbergh@vumc.nl (V.W.M.H.); 2 A-Skin Nederland BV, De Boelelaan 1117, Amsterdam 1007 MB, The Netherlands

**Keywords:** angiogenesis, HIF-1α, HIF-2α, hypoxia, tissue engineering, wound healing

## Abstract

In patients with chronic wounds, autologous tissue repair is often not sufficient to heal the wound. These patients might benefit from regenerative medicine or the implantation of a tissue-engineered scaffold. Both wound healing and tissue engineering is dependent on the formation of a microvascular network. This process is highly regulated by hypoxia and the transcription factors hypoxia-inducible factors-1α (HIF-1α) and -2α (HIF-2α). Even though much is known about the function of HIF-1α in wound healing, knowledge about the function of HIF-2α in wound healing is lacking. This review focuses on the function of HIF-1α and HIF-2α in microvascular network formation, wound healing, and therapy strategies.

## 1. Introduction

Injury causes damage to the blood vessels and thereby an interrupted blood flow. Deprivation of blood supply will rapidly cause tissue hypoxia, a lack of sufficient oxygen to meet the metabolic demand of the tissue. Subsequently, an oxygen gradient will arise between affected and non-affected tissue(s) that stimulates the migration and proliferation of endothelial cells (ECs) and fibroblasts, and adequate angiogenesis to reconstitute normal blood supply [1]. As a result, the wound is revascularized and the oxygen concentration in the wound increases. If this process fails, a prolonged inadequate vascular supply of oxygen leads to chronic hypoxia and can cause non-healing or chronic wounds, such as venous or diabetic ulcers. These wounds generally occur in people older than 60 years of age and are caused by a combination of (1) the cellular and systemic changes of aging; (2) repeated ischemia-reperfusion injury; and (3) a chronic inflammatory environment due to prolonged infection. Acute wounds usually heal in older people without complications, but chronic wounds do not. If tissue repair fails, tissue engineering or regenerative medicine and transplantation is necessary [2].

Tissue engineering, the culture of cells on a biodegradable scaffold *ex vivo*, and implantation has allowed select and limited progress toward the repair of some tissues. The onset of tissue engineering [3] promised the replacement of large or complex damaged organs and regeneration of tissues as bone, liver, skin and blood vessels [4]. However, the development of complex tissues is very complicated and the lack of nutrient and oxygen delivery to and removal of waste products from developing tissues has limited the success rate of tissue engineering so far. Especially the delivery of oxygen to the highly metabolic active cells in the scaffold is a major problem resulting in reduced (hypoxia) or lack of oxygen (anoxia) within the deeper regions of the tissue-engineered scaffold [5,6,7]. Therefore it is important to either prevascularize tissue-engineered scaffolds by creating a blood vessel network in a scaffold *in vitro* or create a scaffold with an environment (matrix composition, incorporation of blood vessel-generating cells and growth factors) that facilitates rapid angiogenesis when implanted in the body. Unfortunately, this has not been realized yet, suggesting that angiogenesis is complicated.

This review focuses on the role of hypoxia and angiogenesis in wound healing and after tissue-engineered scaffold implantation. Angiogenesis is highly regulated by hypoxia and the transcription factors HIF-1α and HIF-2α. Even though many studies and reviews have been published investigated the role of HIF-1α in wound healing [8], less is known about the role of HIF-2α in wound healing.

## 2. Angiogenesis

Blood vessels can be formed through different processes. Vasculogenesis is the formation of blood vessels by endothelial progenitor cells; angiogenesis refers to the sprouting of new blood vessels from existing ones, and subsequent stabilization of these new vessels by mural cells; and arteriogenesis or collateral growth includes the maturation and enlargement of smaller preexisting arterial vessels through vascular remodeling forming collateral bridges between arterial networks.

In the healthy body, endothelial cells (ECs) are quiescent; the cells hardly divide (less than once in 100–300 days), barely form new sprouts, but perform many physiological functions such as barrier between blood and surrounding tissues. Under pathological conditions, endothelial cells are activated by growth factors and inflammatory cytokines such as vascular endothelial growth factor A (VEGF-A), basic fibroblast growth factor (bFGF), platelet-derived growth factor (PDGF), and tumor necrosis factor alpha (TNF-α), and the microvessels can become leaky. As a consequence, plasma proteins like fibrinogen and vitronectin leak from these vessels into the tissue and serve as building blocks for a provisional (fibrin) matrix. At the same time, activated ECs degrade their basement membrane and the extracellular matrix (ECM) through the upregulation and secretion of matrix metalloproteinases (MMPs) and the members of the plasmin-plasminogen activator (PA) pathway. Interestingly, many growth factors are sequestered in the ECM and are released from the ECM by these and other proteases. The ECs develop into a sprouting network of “stalk” cells and a leading “tip” cell. The “tip” cell guides the forming tube that migrates into the tissue toward gradients of chemotactic signals, whereas the proliferating “stalk” cells elongate the tube, form a lumen and form tight junctions. Once the sprout is ready to anastomize with the circulation or an adjacent sprout, a normalized vascular phenotype is induced by inhibiting the proliferation and migration of the endothelial cells. Simultaneously, mural cells including pericytes (in capillaries) and vascular smooth muscle cells (in larger blood vessels) proliferate, migrate, and differentiate, and are recruited to the immature vasculature to provide vessel stabilization and to regulate vessel perfusion. Finally, a basement membrane is formed. The initial wound healing requires a lot of energy and the recruitment of substrates and oxygen, which is met by the formation of many microvessels (the so-called granulation tissue). Once the healing is completed, a selective “pruning” of the vessels occurs, which results in a mature system with larger and smaller vessels [9,10,11,12,13].

### 2.1. Angiogenesis in Tissue Repair

**Figure 1:**
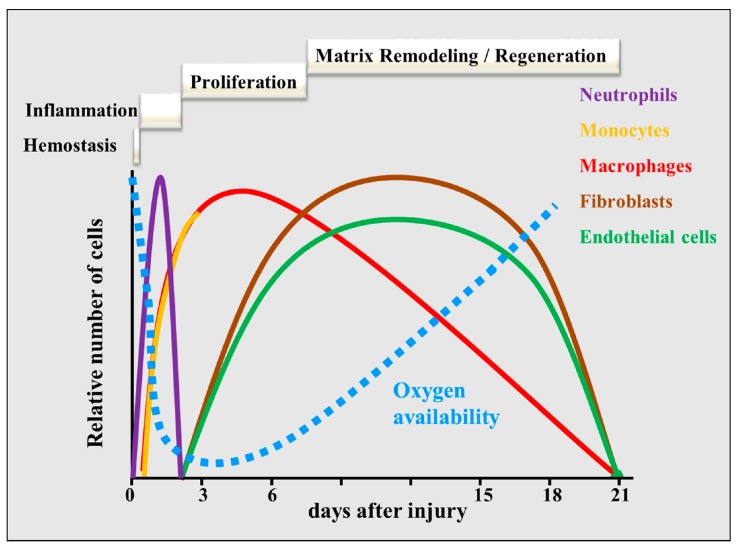
A schematic overview of the phases of wound healing over time. After the initial hemostasis phase, neutrophils and macrophages dominate the inflammation phase, whereas fibroblasts and endothelial cells are predominant during the proliferation phase. During the remodeling phase, fibroblasts and endothelial cells undergo apoptosis or exit the wound. Finally, the granulation tissue and vascular network remodel and mature, which can last for years. The dotted blue line indicates the time course of oxygen availability.

The implantation of a tissue-engineered scaffold results in injury to the tissue, which activates the wound healing cascade. Wound healing and tissue repair consist of a highly organized sequence of complex processes that can be divided into hemostasis, inflammation, proliferation, and remodeling phases [14,15,16,17] (Figure 1). Injury causes damage to the tissue and vessels and therefore the first stage aims at controlling the local bleeding immediately, called hemostasis (seconds to hours after injury). The injured vessels constrict and deliver blood plasma, proteins and blood platelets into the wound site thereby forming a platelet plug. The damaged vessel and platelets activate the coagulation pathway resulting in the formation of a fibrin clot [18]. This fibrin meshwork enforces the platelet plug, by which two important functions are combined: a firm sealing of the damaged blood vessel and the generation of a provisional repair matrix. Indeed, activated platelets release many cytokines including VEGF-A, PDGF, bFGF, and TNF-α [16,19,20,21], which accumulate in the provisional matrix and create a chemotactic gradient that attracts many inflammatory and mesenchymal cells such as neutrophils, monocytes, macrophages, endothelial cells, and fibroblasts [22].

Inflammatory phagocytic cells infiltrate the provisional matrix, and stimulate the inflammation phase (hours to days after injury) to control and remove the injury [23]. Neutrophils (accumulating within 1–2 days post-injury) and monocytes (2–3 days) digest foreign particles, bacteria and dead or dying cells and secrete many pro-inflammatory cytokines [24]. Both the high metabolic demand of the phagocytes and the impaired supply of oxygen to the wound will cause hypoxia within the wound area. Accumulating monocytes differentiate during the inflammation phase into macrophages and these macrophages secrete growth factors and many pro-inflammatory cytokines thereby amplifying the inflammatory response. The macrophages and activated platelets in the wound site release pro-angiogenic factors [22] and induce the proliferation phase (2–10 days), which aims at regenerating the damaged tissue. This phase includes the proliferation and migration of different cell types such as fibroblasts and endothelial cells into the provisional matrix. Furthermore, hypoxic tissue cells surrounding the wound also start producing factors, in particular VEGF, which induces capillary sprouting [25,26] and further attracts monocytes [27]. The angiogenic factors induce ECs forming new capillary-like sprouts, which invade the sealing matrix and the hypoxic wound area. This results in the formation of a microvascular network throughout the wound, providing the healing tissue with nutrients and oxygen. Fibroblasts in close proximity to the wound differentiate into myofibroblasts and invade the wound area. They contract to bring the edges of the wound together [28]. During the remodeling/regeneration phase, a delicate balance between the expansion of microvessels and fibroblasts is needed to avoid scar formation or tissue weakening [29]. Finally, the blood vessels in the granulation tissue mature to form a functional vascular network. The remodeling starts after 1–3 weeks and can last for years [15,16,17].

### 2.2. Vascularization of a Tissue-Engineered Scaffold

The implantation of a cellularized tissue-engineered scaffold runs through a similar sequence of events as wound healing [17]. After implantation, a surrounding fibrin meshwork is formed from the fibrinous exudate around the scaffold. Furthermore, the lack of vascularization will soon cause hypoxia within the scaffold, which within a few days can cause cell death of the whole or the central part of the scaffold [5]. Therefore rapid revascularization is required to preserve the implanted cells. The prerequisites for and sequence of this process are comparable to that of wound healing. Central in this process is the cellular response to oxygen deprivation.

## 3. Cell Signaling after Oxygen Deprivation

Although oxygen represents 21% of ambient air, within the mammalian body the oxygen concentrations vary from 13.5% in the lungs, and 5%–12.5% in the circulation, to 0.6%–5% in tissues [30,31]. Normally, the cardiovascular and respiratory systems ensure an adequate oxygen delivery to cells and tissues. The affected tissue area can become hypoxic [14], due to interrupted blood supply after injury or high oxygen consumptions as occurs in inflammation [5]. This can lead to cellular dysfunction and eventually cell death.

Cells can sense reduced oxygen levels, and respond by altering gene expression in such a way that it conserves energy, promotes cell survival and increases oxygen delivery; both mRNA transcription and protein synthesis is altered [32,33,34]. Many pathways are involved, including Ca^2+^ signaling [35], mTOR and MAP kinase pathways [35,36], internal ribosome entry sites (IRES) [37], and the unfolded protein response (UPR) [32,38]. However, the most prominent mechanism for cells to respond to hypoxic stress is through the transcription factors hypoxia-inducible factors (HIFs).

### 3.1. Regulation of Hypoxia-Inducible Factors

**Figure 2:**
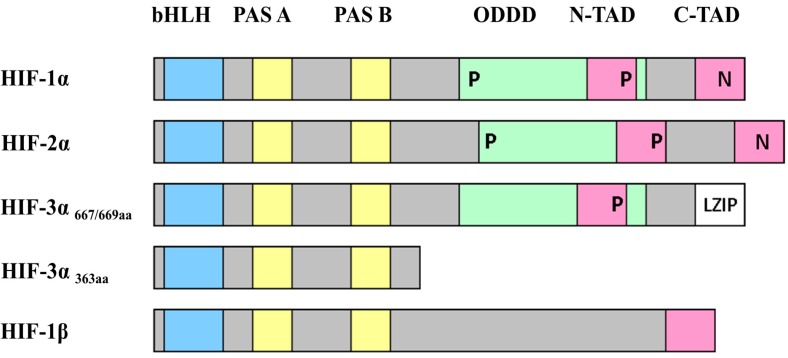
A schematic representation of the domain structures of the HIFs. The figure shows the structural motifs basic-Helix-Loop-Helix (bHLH, in blue), PER/ARNT/SIM (PAS, in yellow), the oxygen-dependent degradation domain (ODDD, in green and also spanning the N-TAD region), the *N*-terminal and *C*-terminal transactivation domains (N-TAD and C-TAD, in pink), and the leucine zipper (LZIP, in white). The positions of the prolines hydroxylated by PHDs (prolyl-hydroxylase domain containing enzymes) are indicated by P and the asparagines hydroxylated by FIH (factor inhibiting HIF) are indicated by N. Adapted from [47].

HIF is a heterodimer consisting of an oxygen-sensitive α-subunit and a constitutively expressed β-subunit [39,40,41]. Both subunits (Figure 2) are part of the basic Helix-Loop-Helix PER-ARNT-SIM (bHLH-PAS) family of transcription factors and these domains are important for DNA binding and dimerization [39]. The HIF-α subunits possess several domains including an oxygen-dependent degradation domain (ODDD) and two transactivation domains (Figure 2). Both the *N*-terminal transactivation domain (N-TAD) and the *C*-terminal transactivation domain (C-TAD) are essential for the regulation of HIF-dependent gene expression [40,42].

Three isoforms of the α-subunit exist in mammals; HIF-1α [43] encoded by HIF1A, HIF-2α [44] encoded by EPAS1, and HIF-3α [45] encoded by HIF3A, which is expressed as three splice variants with different protein lengths (HIF-3α_667aa_, HIF-3α_669aa_ and HIF-3α_363aa_) (Figure 2). Also three paralogues exist of the HIF-β subunit, ARNT1 (aryl hydrocarbon receptor nuclear translocator), ARNT2 and ARNT3, but ARNT1 is the primary HIF-1β subunit involved in the hypoxic response [46]. In the remaining chapters HIF-α refers to HIF-1α and HIF-2α and HIF-1β refers to ARNT1.

**Figure 3:**
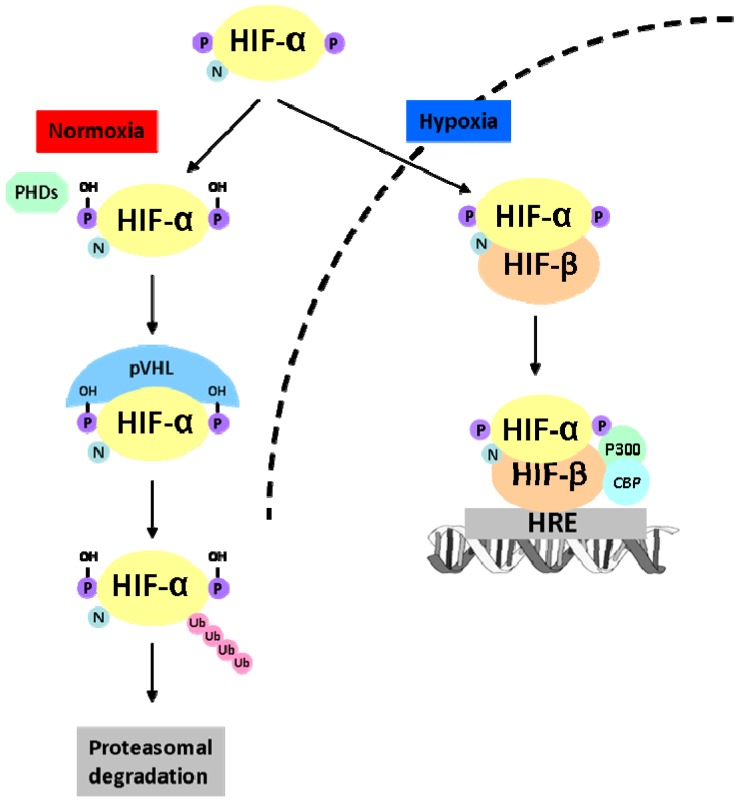
A schematic representation of the oxygen-dependent regulation of HIF-α. During normoxia, HIF-α is hydroxylated by prolyl hydroxylase domain proteins (PHDs) on proline residues. These proline residues are recognized by the protein Von Hippel Lindau (pVHL), which results in proteasomal degradation. Upon hypoxia, HIF-α is not hydroxylated and subsequently translocated to the nucleus. In the nucleus, HIF-α heterodimerizes with HIF-β; this allows the co-factors p300 and Creb-binding protein (CBP) to bind to the heterodimer. The complex binds to the hypoxia-responsive elements (HRE) and thereby induces gene expression. Adapted from [60].

The HIF-α subunit is constitutively transcribed and translated, but has an extremely short half-life of 5 min due to constitutive degradation of the protein [39,41]. Under normal conditions (normoxia), when oxygen is present, (Figure 3), HIF-α is hydroxylated on two proline residues in the ODDD by prolyl-hydroxylase domain containing enzymes (PHDs) and in the C-TAD on an asparagine residue by factor inhibiting HIF (FIH) (Figure 2) [48,49]. The proline residues are located in the conserved amino acid sequence LxxLAP; two of these sequences exist in HIF-1α (Pro402 and P564) and HIF-2α (Pro405 and Pro531), and one sequence exists in HIF-3α (Pro490). The hydroxylated proline residues form a binding site for the von Hippel Lindau tumor suppressor protein (pVHL), an E3 ubiquitin ligase, which results in ubiquitination of HIF-α and its subsequent degradation by the proteasome [49,50]. Hydroxylation on the asparagine residue Asn803 (for HIF-1α) and Asn847 (for HIF-2α), blocks the interaction of HIF-α with the co-factors p300 and Creb-binding protein (CBP), and thereby inhibits the transcription of C-TAD regulated genes. Both PHDs and FIH belong to the evolutionary conserved α-ketoglutarate dependent Fe(II)-dioxygenases that require iron and ascorbate as co-factors to insert one oxygen atom into prolyl residues and the other oxygen atom to split α-ketoglutarate into succinate and CO_2_ [48,51].

During hypoxia, the HIF-α subunit escapes hydroxylation and thereby degradation (Figure 3). Subsequently, HIF-α translocates to the nucleus where it forms a heterodimer with HIF-β and this complex binds to the hypoxia-responsive elements (HREs), which contain the core sequence 5'-(A/G)CGTG-3' of the HIF-responsive genes [43,52]. These genes regulate processes such as proliferation, apoptosis/autophagy, DNA damage response, extracellular matrix metabolism, cell migration and invasion, survival, metabolism, inflammation, and an increase of oxygen delivery via erythropoiesis and angiogenesis [53,54].

The HIF-α subunit can also be stabilized under normal oxygen conditions or by pharmacological tools despite normal oxygen availability. Inhibition of the enzymatic function of PHDs by nitric oxide, reactive oxygen species (ROS), iron chelators and several metabolic intermediates of the tricarboxylic acid (TCA) cycle such as succinate and fumarate can stabilize HIF-α [48,51,55]. Furthermore, loss-of-function mutations of pVHL, which can be present in specific kidney tumors, can cause stabilization of HIF-α [50]. Finally, HIF-α can be stabilized through the activation of the phosphatidylinositol-3-kinase (PI3K), MAP kinase and phospholipase Cγ (PLCγ) pathways upon binding of growth factors and cytokines such as epidermal growth factor (EGF) and insulin-like growth factor-1 (IGF-1) [56,57] to their tyrosine kinase receptors or G-protein coupled receptors. Moreover, proteins in the MAPK pathway can directly phosphorylate HIF-1α [58,59], but it is not known whether this influences HIF-1α-induced gene transcription.

### 3.2. Function of Hypoxia-Inducible Factors

HIF-1α and HIF-2α-induced gene expression under hypoxia share many similar target genes, but also have many unique target genes [61,62,63]. HIF-1α and -2α regulate similar genes synergistically and can partly compensate each other in ECs during embryonic development. The C-TAD and N-TAD contribute to the regulation of gene transcription and both are necessary to optimally induce gene expression [63]. The C-TAD is highly homologous between HIF-1α and HIF-2α and promotes the expression of HIF-1α/HIF-2α common genes while the N-TAD is less homologous and thus important for target gene specificity [63]. The HIF-3α splice variants are also homologous to HIF-1α and -2α, but lack the C-TAD, and the splice variant HIF-3α_363aa_ also lacks the N-TAD (Figure 2). Therefore, HIF-3α_363aa_ cannot induce gene expression and is thought to be an inhibitor of HIF-1α and -2α-induced gene expression. The splice variants HIF-3α_667aa_ and HIF-3α_669aa_ can induce gene expression, but only very weakly [45,64,65].

Although HIF-1α and HIF-2α respond to similar stimuli in the cell, they often control different pathways. A central adaptation to hypoxia is the shift toward anaerobic glycolysis. HIF-1α guides this shift by promoting the expression of glucose transporters and glycolytic enzymes [62,63,66,67]. Moreover, during hypoxia, the mitochondria release ROS, which can cause DNA damage. HIF-2α suppresses aberrant ROS accumulation by regulating the expression of antioxidant enzymes, such as superoxide dismutase-2 (SOD2) and heme oxygenase 1 (HMOX1) [68,69]. Moreover, HIF-1α and HIF-2α have antagonistic effects on adult and embryonic angiogenesis [70], which will be discussed in detail below.

## 4. HIFs and Angiogenesis

### 4.1. HIF-1α

Prenatal knockout of HIF1A in mice is embryonic lethal due to neural, cardiac and angiogenic defects [67,71,72,73]. The HIF1A^−/−^ embryos appear normal at day E8.5, but at day E9.5 these embryos display vascular defects such as inadequate vessel formation and abnormal large endothelial-lined vascular structures that are not rescued by HIF-2α [67,74]. The embryonic yolk sacs completely lack organization in the branching of the vasculature, although the vessels are fully formed containing erythrocytes [72]. Also, the dorsal aorta and atrium contain erythrocytes in HIF1A null embryos [73].

HIF1A^+/−^ heterozygote mice are born according to a normal Mendelian ratio and appear normal and healthy with normal hematocrit levels [67,71,75], but the mice display impaired pulmonary adaptation to chronic hypoxia [75].

Interestingly, endothelial-specific HIF1A-deficient mice develop normally, suggesting that for proper vascularization during embryonic development, interaction with non-endothelial cells is pivotal. However, these mice could barely form vessels in tumors or in a repair setting *in vivo*. Notwithstanding, erythrocytes were visible in the few vessels that developed, suggesting that those vessels were functional [61]. In line with an impaired angiogenesis in a pathological setting, under reduced oxygen conditions, the proliferation, migration, invasion and tube formation was hampered in HIF1A-deficient endothelial cells [61,76] or embryonic stem cells [67]. Moreover, HIF1A null endothelial cells could not form tubular networks in hypoxia *in vitro* [61].

Clearly, HIF-1α stimulates processes involved in vessel sprouting and neovascularization such as endothelial cell proliferation, migration, and tube formation.

### 4.2. HIF-2α/EPAS1

While HIF-2α (also called EPAS1, endothelial PAS domain containing protein 1) is expressed more selectively than HIF-1α, its expression in endothelial cells is relatively strong [44]. It is also encountered in several other cell types, such as hematopoietic cells [77] and keratinocytes [78].

The generation of HIF2A^−/−^ mice resulted in embryonic or perinatal lethality, but the severity of vascular defects depended on the genetic background of the mice. HIF2A^−/−^ embryos intercrossed between mice with the same genetic backgrounds showed severe vascular defects and died before day E12.5 [68,79,80]. However, HIF2A^−/−^ embryos intercrossed from different mouse strains showed less severe vascular defects, but the degree of the defects varied, probably due to compensating mechanisms [68,77,79,81]. The yolk sacs of HIF2A^−/−^ embryos showed misarranged endothelial cells and smaller blood vessels that merged into extensive endothelial sheets. Some organs showed normal vascular development but in other organs the small vessels failed to remodel into larger vessels. And if large vessels were formed, they were leaky. Re-expression of HIF-2α in endothelial cells in HIF2A^−/−^ embryos could rescue 30% of the embryos beyond day E12.5 and large vessels were observed that branched into progressively smaller sprouts [80].

Postnatal whole body HIF2A deletion also resulted in reduced erythrocyte levels, hematocrit values and hemoglobin levels leading to anemia. Moreover, hematopoietic progenitor cells had reduced differentiation levels in postnatal HIF2A^−/−^ mice [82].

The HIF2A^+/−^ heterozygote mice were born according a Mendelian ratio and showed no vascular defects in the heart, kidneys or retina and had normal hematocrit levels [77,79,83,84], but the retinas in HIF2A^+/−^ mice showed reduced neovascularization after oxygen-induced retinopathy [84].

Endothelial cell-specific HIF2A^−/−^ mice exhibited increased acute vascular leakage in response to VEGF stimulation and defective tumor neovascularization [70]. HIF-2α-deficient endothelial cells display increased migration, invasion and tube formation, reduced adhesion, but no difference in proliferation or viability *in vitro* under hypoxic conditions [70,76,79]. Also, more new vessels were formed in endothelial-specific HIF2A^−/−^ mice, these vessels failed to remodel into mature, functional blood vessels *in vivo* [76].

Clearly, HIF-2α is involved in restricting angiogenesis-related processes like endothelial cell migration and sprouting and promotes vessel remodeling into mature, functional vessels.

### 4.3. HIF-3α

HIF-3α consists of three human splice variants HIF-3α_667aa_ (in mouse HIF-3α), HIF-3α_363aa_ (in mouse IPAS), and HIF-3α_669aa_ (in mouse NEPAS), which are homologous to HIF-1α and HIF-2α, but they cannot induce gene expression or only very weakly [45,64,65].

Moreover, HIF-3α mRNA is increased after exposure to hypoxia, suggesting that the HIF-3α variants are targets of HIF-1α-induced gene expression [85]. Both HIF-3α_667aa_ and HIF-3α_363aa_ are not detectable in embryos [64], but are expressed in adult stages e.g. in the brain and eyes [45]. HIF-3α_669aa_ is exclusively expressed in the late embryonic stages and early postnatal stages with the highest expression in the vessels and the heart [64].

Cells expressing HIF-1α and HIF-2α and one of the HIF-3α variants show decreased levels of hypoxia-induced gene expression [45,64,65]. All the HIF-3α splice variants can bind directly to HIF-1α, HIF-2α and HIF-1β. The binding of HIF-3α to HIF-1α prevents the translocation of HIF-1α to the nucleus [65]. Moreover, the binding of HIF-3α to HIF-1α, HIF-2α or HIF-1β inhibits the heterodimerization of HIF-1α-HIF-1β or HIF-2α-HIF-1β and thereby HIF-1α/HIF-2α-mediated gene expression. Overexpression of HIF-1β reversed the HIF-3α-mediated suppression of gene expression [64]. Apparently, the HIF-3α splice variants function as dominant negative regulators of HIF-1α and HIF-2α-mediated gene expression when HIF-1β is limiting.

HIF-3α_667aa/669aa_^−/−^ mice were born according a Mendelian ratio. These mice appeared normal, were viable and fertile, but lung formation was delayed and lung remodeling was impaired. Moreover, the right atrium and ventricle was slightly enlarged, but the myocardium contained many microcapillaries [64].

Epithelial cells in the cornea, an avascular and hypoxic tissue, express high concentrations of HIF-3α_363aa_ but hardly any HIF-1α. Silencing HIF-3α_363aa_ in the cornea induces neovascularization [45].

These data suggest that the splice variants of HIF-3α function as dominant negative regulators of HIF-1α and HIF-2α by binding to HIF-1α, HIF-2α and HIF-1β. However, the HIF-3α splice variants are not global repressors of the hypoxia-induced gene response, but function more locally in various tissues such as the cornea.

### 4.4. HIF-1β/ARNT

Whole body ARNT^−/−^ embryos are embryonic lethal and die before day E10.5 due to defects in angiogenesis, cardiogenesis, hematopoiesis and placentation [86,87,88,89,90]. The embryos exhibited improper vessel formation, remodeling and maturation. The yolk sacs of ARNT^−/−^ embryos at day E8.5 appeared normal and had a normal vascular development. At day E9.5 most of the embryonic yolk sacs contained no vasculature and other yolk sacs contained some vessels that were not filled with blood. Moreover, the yolk sacs showed defective endothelial remodeling; the endothelial cells were fused with each other and the few capillaries that were present in the yolk sacs fused into enlarged structures [87,90]. Also the heart and dorsal aorta contained less endothelial cells resulting in a disorganized vascular network.

ARNT^+/−^ heterozygote mice were born according a Mendelian ratio and all pups were viable [86,90].

Ninety percent of EC-specific ARNT^−/−^ embryos died prenatal [91] and these embryos had no blood in their umbilical cords or placentas. Although the hearts and livers of these embryos showed lesions with hemorrhages and contained more areas lacking vasculature compared with wild-type embryos, the development and functioning of most organs were not affected by ARNT deficiency in endothelial cells. Surprisingly, the live EC-specific ARNT^−/−^ embryos and their yolk sacs were indistinguishable from wild-type embryos. These mice were fertile and appeared normal.

These data suggest that HIF-1β seems to be involved in embryonic and yolk sac angiogenesis, but not in vasculogenesis.

## 5. HIF-1α and HIF-2α Expression During Wound Healing

There is much known about the role of HIF-1α in wound healing. However, recently a paper was published investigating the role of HIF-2α in wound healing.

HIF-1α expression is increased after tissue injury through hypoxia-dependent and -independent mechanisms. Immediately after injury, even before detectable hypoxia, TNF-α induces HIF-1α expression in primary inflammatory cells [92,93]. In the later inflammatory phase, hypoxia is present and stabilizes the HIF-1α protein [93]. Moreover, during the inflammatory response, HIF-1α and HIF-2α play important roles in the secretion of pro-inflammatory cytokines in macrophages [94,95].

In animal models, the effect of HIF-1α in multiple wounding models was examined. During spontaneous wound healing in the skin, HIF-1α was abundantly expressed in keratinocytes invading the provisional matrix and re-epithelializing the wound and HIF-1α is involved in the formation of new capillary sprouts invading the wound clot [61,96]. Moreover, in burned tissue, HIF-1α protein levels increased, resulting in the mobilization of circulating angiogenic cells, and in vessel formation at the healing margin of granulation tissue [97,98]. Burn wounds in mice with HIF1A deletion exhibited delayed wound closure and reduced vascularization [99]. During acute gastric mucosal injury and esophageal ulcer induction, mainly the endothelial cells adjacent to the necrotic tissue of the wound bed and regenerating microvessels express increased HIF-1α protein levels [100,101].

Chronic wounds are characterized by persistent inflammation, lack of proliferative cells, and increased proteolytic activity preventing sufficient extracellular matrix deposition [102]. Reduced levels of HIF-1α protein have been documented in a number of chronic conditions associated with impaired wound healing including diabetes and aging [98,103,104]. In diabetic wounds, high glucose concentrations impair both HIF-1α stabilization via VHL-mediated degradation although HIF-1α mRNA is expressed [103,105,106] and HIF-1α induced gene transcription by influencing the activity of the N-TAD and C-TAD. Therefore, despite the increased hypoxia in diabetic wounds, HIF-1α protein levels are decreased and transcriptional activity is reduced. Moreover, during aging, PHD levels are upregulated leading to increased HIF-1α degradation, even under hypoxic conditions [104].

Surprisingly, the impaired wound healing in diabetic or aged wounds seems to be reversible; upon over-expression of a constitutively active form of HIF-1α, HIF-1α protein levels were increased in the granulation tissue, and the epithelial regeneration and angiogenesis in both the wound bed and proximal skin were improved. Moreover, the number of total vessels was increased and the overall diabetic wound healing was improved [103,105,107,108].

HIF-2α protein was stabilized at the wound margin in full-thickness dermal wounds with the highest expression after 5 days. Surprisingly, in contrast to HIF-1α, HIF-2α probably impairs wound healing since HIF2A^−/−^ in keratinocytes showed increased migration and wound healing [78]. In addition, endothelial-specific deletion of HIF2A in mice resulted in increased sprouting, but less functional vessels [76].

## 6. Tissue Repair/Tissue Engineering

Comparable to healthy tissue, injured tissue and tissue-engineered scaffolds require blood vessels for their oxygen and nutrient delivery and waste product removal. However, oxygen diffusion is limited in a cellular tissue-engineered scaffold [7,109], and cells located more than 100 μm from the nearest capillary blood vessel experience hypoxia [5,6]. Therefore, new microvascular network formation is a critical component of wound healing and tissue regeneration [110,111,112,113]. Moreover, blood vessels transport signaling molecules such as angiogenic factors to stimulate endothelial sprouting during wound healing and reestablish a connection from the avascular zone to the circulation.

### 6.1. Therapeutic Angiogenesis

Angiogenesis is dependent on a proper balance between pro- and anti-angiogenic factors [9] and between sprout-inducing and -inhibiting factors [13]. An imbalance between these factors will cause chaotic, irregular, and leaky vessels with many avascular and hypoxic areas, a situation often observed in tumors [114,115,116]. Tumor cells proliferate fast and therefore tumors frequently have hypoxic areas. These hypoxic areas stimulate tumor angiogenesis and the newly formed vessels are involved in the metastatic characteristics of tumors. Moreover, the hypoxic environment and leaky vessels counteract tumor therapy; chemotherapy cannot be delivered to the tumor due to poor perfusion of the vessels [116] and oxygen is required for the cytotoxic effect of ionizing radiation, which is inhibited in hypoxic environments [117]. Therefore, a delicate balance between stimulating and inhibiting angiogenesis is very important.

#### 6.1.1. Inhibiting Angiogenesis

Therapeutic inhibition of angiogenesis appears to be very complicated. Inhibiting angiogenesis was thought to be a treatment for tumors. Even though in different tumor mouse models blocking VEGF or the VEGF receptor 2 (VEGFR2 or KDR) with inhibitors was shown to effectively reduce tumor vasculature or disrupt the existing vasculature, the tumors were more aggressive and invasive [114,118]. And after the treatment was stopped, the tumor vessels regrew to the same number and irregularity as before treatment [114].

Although wound healing requires angiogenesis [113], excess connective tissue density and excessive newly formed vessels could reduce the overall quality of the healed wound, as demonstrated by the use of a broad-spectrum angiogenesis inhibitor [119].

#### 6.1.2. Stimulating Angiogenesis

The stimulation of angiogenesis is more complicated than initially thought. The formation of initial sprouting during angiogenesis is highly stimulated by growth factors and the majority of these factors are stimulated by hypoxia and the HIFs, which induce, amongst others, the potent angiogenesis stimulator VEGF-A [9,120]. Hypoxia and the HIFs stimulate processes such as endothelial cell survival, proliferation, capillary sprouting and the directed migration of endothelial cells toward a chemotactic gradient, as well as the recruitment of pericytes and the eventual inhibition of proliferation and migration once a functional sprout has been formed [9,10,12].

Injection of high concentrations of VEGF induced tortuous and leaky newly formed vessels in rodents [121,122]. Injection of high concentrations of VEGF, however, was not sufficient to induce neovascularization in the retina of primates [121]. Moreover, treatment of wounds with VEGF did not improve wound healing, and high concentrations even impaired wound healing [123].

It is thought that the usage of autologous stem cells could be beneficial for repairing or regenerating injured tissues. Recently, Sánchez Muñoz *et al.* [124] showed in an artificial skin substitute model of fibroblasts and keratinocytes embedded in a fibrin matrix that capillary-like structures were only formed when human umbilical cord endothelial cells were implanted in the fibrin matrix in combination with human adipose-derived mesenchymal stem cells. These capillary-like structures were lined with CD31^+^ and vWF^+^ cells, suggesting that these lining cells are endothelial cells.

### 6.2. Angiogenesis and Hypoxia as Therapeutic Tools in Tissue Engineering and Tissue Repair

A prevascularized scaffold requires the isolation of cells from the patient, followed by *in vitro* expansion and conditioning of cells within the scaffold before implantation. However, stimulating the formation of a vascular network *in vitro* is complicated. It was thought that endothelial cells cultured in a matrix in the presence or absence of angiogenic factors *in vitro* could generate a primitive vascular network that would be sufficient to vascularize the scaffold. Although the primitive vascular network was formed and could form anastomoses with the host vessels [125,126], the engineered vessels were only functional upon introduction of anti-apoptotic genes [110]. During the transfer of a prevascularized tissue-engineered scaffold from an oxygenated *in vitro* environment to a less oxygenated *in vivo* implantation site, the oxygen level drops. Moreover, the generated vascular system has to connect to the vascular network of the patient soon after implantation to maintain viability of the seeded cells [113]; the cells on the outside of the tissue-engineered scaffold consume high amounts of oxygen resulting in an oxygen gradient from the outside toward the inner part of the scaffold, where almost no oxygen is available [5,6,109]. The result is cell death and degradation of the non-perfused capillary network [5,127].

A similar oxygen gradient is present in injured tissues and this oxygen gradient stimulates the expression and secretion of cytokines and growth factors and therefore the migration and proliferation of endothelial cells and fibroblasts. Moreover, epithelialization is increased in hypoxia and angiogenesis is stimulated [1,25,128,129] leading to revascularization in the newly formed granulation tissue and subsequent wound healing. Therefore, it has been proposed that short-term exposure to hypoxia stimulates tissue repair and angiogenesis [130].

However, if the decreased oxygen levels persist inside the scaffold, the cells will die. Prolonged severe hypoxia decreases the proliferation rate and granulation tissue synthesis in fibroblasts *in vitro*, causes vascular degeneration, and eventually endothelial cells will undergo apoptosis [102,128]. Moreover, clinical animal studies have shown that wound healing is delayed under prolonged hypoxia and low oxygen levels were found in non-healing chronic wounds. The delay in wound healing was due to reduced granulation tissue production and delayed epithelialization [131,132,133], and lack of oxidants required for the prevention of wound infection [130,134,135,136]. *In vivo*, oxygen levels between 0.5%–1.5% were measured at the threshold between the epicenter of the wound and the granulation tissue; leukocytes were observed in this area whereas proliferating fibroblasts were only found in areas with higher oxygen levels.

### 6.3. HIF-1α and HIF-2α as Therapy?

As mentioned above, tissue repair and tissue engineering require the formation of a mature vascular network and a proper connection to the host vessels. The induction of a mature vascular network is dependent on a precise balance of many pro- and anti-angiogenic factors. In this induction, HIF-1α and HIF-2α play in part different roles. Amongst many functions, HIF-1α is a strong inducer of capillary sprout formation in particular by inducing VEGF-A transcription in all cells [26]. HIF-2α has more of a balancing role, not only by a more selective expression, but in particular because in endothelial cells it induces sprout stabilization [76] and its VEGF-inducing capacity is weaker than that of HIF-1α [137].

Overexpression of HIF-1α by gene transfer [138] or by blocking HIF-1α degradation [139,140,141,142,143] can stimulate angiogenesis and non-leaky vessels in multiple animal studies. Reduced levels of HIF-1α impair angiogenesis [97]. Interestingly, overexpression of HIF-1α in chronic wounds in diabetic or aged mice improved the healing, granulation tissue formation, and angiogenesis in both the wound bed and proximal skin [103,105,108]. The iron chelator deferoxamine increased the binding of HIF-1α to its co-factor p300 and thereby normalized the high-glucose induced reduction in VEGF expression. This resulted in increased neovascularization, granulation tissue formation and enhanced wound healing rate in diabetic mice [107]. Therefore topical application of deferoxamine might be a potential candidate for improving wound healing in diabetic patients that should be further evaluated.

On the other hand, consistent increased levels of HIF-1α, but not HIF-2α, are associated with hypertrophic scars and system sclerosis. These scars express increased levels of profibrotic and growth factors and contain many hyperproliferative cells, excessive deposition of collagen and increased vasculature [144,145]. Furthermore, HIF-1α is highly expressed in many tumors due to hypoxia-dependent and -independent mechanisms and is correlated with tumor growth and a more aggressive tumor phenotype [53,146,147]. Enhancement of HIF-1 activity might therefore be counterindicated in tumor or tumor-prone individuals. Indeed, several HIF-1 inhibitors are in clinical trials [148], and these inhibitors reduce tumor vascularization and tumor growth in mice [149]. On the contrary, selective HIF-1 modulating agents, like PHD inhibitors already receive (clinical) interest as a means to increase erythropoietin production in the kidney and subsequent erythropoiesis [150,151].

HIF-2α was expressed in wounds with the highest expression a couple of days after wounding. This suggests an involvement in wound repair. Reducing HIF-2α expression in keratinocytes increased the rate of wound healing [78]. Moreover, mice with an endothelial cell-specific HIF2A deletion produced increased numbers of small vessels and capillaries but failed to remodel them into mature, functional blood vessels. In addition, deletion of delta-like 4 (Dll4), a target gene of HIF-2α, showed an increase in capillary formation but these capillaries were disorganized [76]. These observations are compatible with a capillary-stabilizing role of HIF-2α [76]. This makes HIF-2α a potentially attractive target for modulating angiogenesis in tissue repair.

HIF-2α can be modulated by direct stimulation of its expression or indirectly by influencing factors that modulate HIF-2α expression. For example, EC-specific deletion of KEAP1 (Kelch-like ECH-associated protein 1) in mice reduced the hypoxia-induced HIF-2α and Dll4 expression, while it increased Nrf2 (NF-E2–related factor 2) expression in murine retinas. The KEAP1^−/−^ retinas showed hypersprouting, similar as observed when Dll4 was blocked in wild-type retinas. Silencing of KEAP1 also increased HIF-1α expression in hypoxia, suggesting that silencing KEAP1 disturbs the balance between HIF-1α and HIF-2α toward sprouting [152]. An interesting alternative approach was reported by Chen *et al.* [153], who silenced the tumor suppressor Int6/eIF3e in normoxic mice. Silencing of Int6/eIF3e resulted in a 2-fold increased expression of HIF-2α, without affecting HIF-1α. Mice injected with siRNA-Int6 showed enhanced neovascularization and excisional wound healing both in control and diabetic mice. This increase in neovascularization was completely abolished by silencing HIF-2α simultaneously, suggesting that this improvement was dependent on HIF-2α. In this particular condition, HIF-2α stimulated both vascular sprouting and stabilization.

Before translation to humans can be made, additional aspects need to be taken into account, in particular regarding the use of HIF-modulating agents in chronic wounds. One has to consider that in advanced diabetes patients often the proximal arteries have also been affected, which interferes with an adequate blood supply to the chronic wound and may require additional treatment. Mice with an EC-specific HIF-2α deletion display less collateral arteries and smooth muscle recruitment to the collateral arteries after femoral artery ligation [76] indicating that HIF-2α also plays a role in arterial remodeling and improving blood supply. Moreover, albeit HIF-1α is highly expressed in limb muscles exposed to short-term hypoxia, where it can function as a stimulator of wound healing, in limb muscles with chronic hypoxia, HIF-1α is hardly expressed [154]. This indicates that a precise balance between HIF-1α and HIF-2α is necessary for a proper induction of sprouting and maturation of blood vessels.

## 7. Conclusions

Almost every stage of normal wound healing is influenced by hypoxia and HIFs. In response to hypoxia, HIF-1α and HIF-2α are stabilized and induce the expression of many downstream target genes involved in many processes including angiogenesis, proliferation and cell survival. HIF-1α stimulates angiogenic processes involved in vessel sprouting and neovascularization whereas HIF-2α promotes vessel remodeling into mature, functional vessels. Deficiency of HIF-1α can lead to non-healing chronic wounds whereas overexpression of HIF-1α can improve wound healing but could also induce hypertrophic scars and tumor growth. HIF-2α impairs wound healing and deficiency of HIF-2α improves wound healing. Modulation of HIF-1α or HIF-2α expression through both positive and negative regulators may provide a promising therapeutic to improve wound healing. However, the complexity of the HIF signaling pathway and existence of multiple HIF and PHD isoforms present a continuing challenge to the development of clinically effective targeted HIF therapies. Manipulation of specific downstream targets of HIF-1α and HIF-2α might support improved wound healing in chronic hypoxic wounds and the success of tissue-engineered scaffold implantation.
